# Relationship between distribution patterns of Qinghai spruce populations in the Qilian mountains and environmental factors

**DOI:** 10.3389/fpls.2026.1834014

**Published:** 2026-06-23

**Authors:** Xiurong Wu, Erwen Xu, Jingzhong Zhao, Wenmao Jing, Shunli Wang, Xuee Ma, Hao Yuan, Nan Zhao, Bin Wang, Ariunaa Ochir, Peifang Chong

**Affiliations:** 1 Academy of Water Resource Conservation forests of Qilian Mountains in Gansu Province, Zhangye, Gansu, China; 2 Qilian Mountains Forest Ecosystem Positioning Observation and Research Station, Zhangye, China; 3 Mongolia Plant Protection Research Institute, Ulaanbaatar, Mongolia; 4 Forestry College of Gansu Agricultural University, Lanzhou, China

**Keywords:** distribution patterns, ecological restoration, environmental factors, Qinghai spruce (*Picea crassifolia*), remote sensing

## Abstract

The Qilian Mountains form a critical ecological barrier in western China, with Qinghai spruce (*Picea crassifolia*) forests as the dominant forest community. Understanding the environmental factors controlling their distribution is essential for forest conservation and ecological restoration under climate change. We combined remote sensing data, DEM-derived topographic variables, long-term meteorological records, and field soil surveys to investigate the distribution patterns of Qinghai spruce in the Dayekou watershed of the Qilian Mountains. Decision tree and random forest models were applied to assess the relative importance of environmental factors. Elevation was the most important topographic predictor (weight = 0.52), followed by slope aspect (0.35), while slope gradient had a weaker influence (0.13). Qinghai spruce primarily occurred between 2900 and 3300 m, favoring shaded to semi-shaded slopes, particularly north-, northeast-, and northwest-facing aspects, which together accounted for 69.73% of the mapped forest area. Under the elevation-corrected framework, the observed distribution corresponded to an annual mean temperature of -3.03°C to 1.45°C and annual precipitation of 394.62–574.69 mm. Soil depth was positively correlated with population density, with denser populations on deeper soils. These findings indicate that Qinghai spruce distribution in the watershed is shaped by the combined effects of topography, hydrothermal conditions, and soil depth. The quantified environmental ranges provide practical guidance for afforestation and ecological restoration in arid mountain watersheds with similar conditions.

## 1 Introduction

Qinghai spruce is a tree species endemic to western China, primarily distributed across Qinghai, Gansu, Ningxia, and Inner Mongolia, with its core area centered in the central-eastern Qilian Mountains. It is a dominant species in the forests of the Qilian Mountains, where it forms nearly pure spruce forests in some areas. Additionally, it plays a critical role in water-conserving forests, performing irreplaceable ecological functions, including maintaining biodiversity, preserving water resources, and controlling soil erosion (Chang et al., 2014). However, climate change and human activities are causing the restructuring of environmental gradients in mountainous regions, thereby threatening the spatial adaptability of tree species (Wang and Yang, 2021; Fang and He, 2020). Understanding the coupling mechanisms between Qinghai spruce distribution and environmental factors has become the scientific foundation for assessing forest vulnerability and formulating conservation strategies (Wang et al., 2020).

The Qilian Mountains represent one of the primary distribution areas for Qinghai spruce, characterized by complex topography, climate, and ecological conditions. Previous scholars’ studies on the distribution characteristics of Qinghai spruce and its relationship with environmental factors has predominantly concentrated on climate change, soil conditions, topography, and biological traits. The prevailing conclusion is that the growth and distribution of Qinghai spruce are influenced by a combination of environmental factors, with climate, soil, and topography being the primary determinants (Wang et al., 2016; Tian et al., 2017). The distribution range of Qinghai spruce is influenced by precipitation and temperature. Within specific watersheds of the Qilian Mountains, factors such as elevation, aspect, and slope gradient limit the distribution of Qinghai spruce forests (Yang et al., 2017). Existing research has mainly focused on the individual effects of climate, soil, and topography on the growth of Qinghai spruce, or has been limited to localized sample plots. However, quantitative studies examining the synergistic interactions among these environmental factors—particularly how their changes drive the fragmented expansion of Qinghai spruce forest distribution boundaries and quantify their respective contributions—are still lacking (Wang et al., 2017; Gao et al., 2021). High-resolution remote sensing has revealed that Qinghai spruce forests have experienced fragmented expansion over the past 50 years. However, the driving mechanisms behind environmental factor changes influencing distribution boundaries remain unquantified. While remote sensing analysis covers large areas, it lacks ground verification, and the two approaches have not been effectively integrated. Few studies have quantitatively combined environmental data with remote sensing technology to create a comprehensive spatial distribution model for Qinghai spruce forests (Tian et al., 2017; Qin et al., 2024; Gao et al., 2025).

In this study, the Dayekou watershed in Qilian Mountain National Park was selected as a representative watershed-scale case to investigate the distribution pattern of Qinghai spruce. By combining full-coverage remote-sensing interpretation, DEM-derived topographic information, long-term meteorological records, and field-based soil observations, we quantified how elevation, slope aspect, slope gradient, temperature, precipitation, and soil depth are associated with the current spatial distribution of Qinghai spruce forests. Compared with previous studies focused mainly on individual plots or single environmental gradients, the specific contribution of this study lies in linking watershed-scale forest-pattern mapping with field information and machine-learning analysis, thereby providing a more spatially explicit assessment of the relative roles of topography, climate, and soil conditions. The results are intended to support forest management, ecological restoration, and a broader understanding of forest-environment relationships in high- elevation arid mountain systems.

## 2 Methods and datasets

### 2.1 Study area

**Figure 1:**
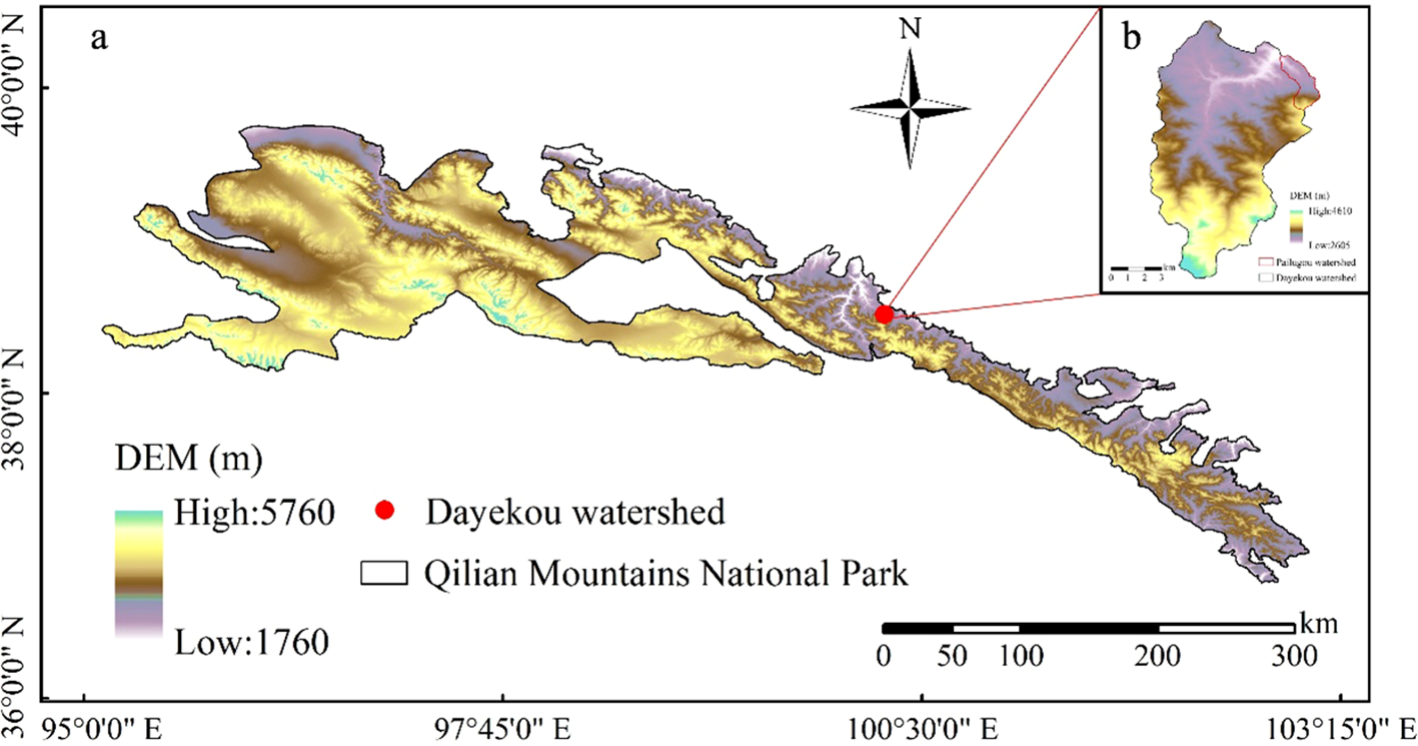
Geographic location map of the study area. **(a)** DEM map of the Qilian Mountains, with red solid circles marking the Dayekou watershed; **(b)** DEM map of the Dayekou watershed, with red boxes delineating the boundary of the Pailugou watershed.

The study area is located in the Dayekou watershed (nested within the Pailugou watershed) on the middle northern slope of the Qilian Mountain National Park. Situated in the middle and upper reaches of China’s second-largest inland river, the Heihe River, its geographical coordinates are E 100°13′–100°16′, N 38°16′–38°33′ (**Figure 1**). The Dayekou watershed covers approximately 72 km² with elevations ranging from 2605 m to 4610 m. The nested Pailugou sub-watershed spans 2.74 km² at elevations between 2650 m and 3800 m (Xu et al., 2024; Wang et al., 2024). The watershed exhibits complex and diverse topography, belonging to the northern Qilian Mountain fold belt with high-mountain deep-valley landforms. Its climate is classified as a temperate continental high-elevation semi-arid, semi-humid mountain climate, featuring an annual average temperature of -0.6 to 2.0 °C. Annual precipitation ranges from 300 to 500 mm, primarily concentrated between June and September. Annual evaporation amounts to 1488 mm, with an average annual relative humidity of 60% (Chang et al., 2025; Wang et al., 2025).

Vegetation distribution within the watershed displays distinct vertical zonation patterns, influenced by elevation gradients and hydrothermal conditions. From lower to higher elevations, the vegetation transitions through four major ecological zones: montane grassland, montane forest-grassland, subalpine meadow, and alpine glacial vegetation (Du et al., 2024). The dominant tree species in the watershed is Qinghai spruce, found in patches or blocks on shaded and semi-shaded slopes at elevations between 2600 and 3300 meters. These stands are derived from natural secondary forests with relatively low disturbance levels, primarily due to human activities such as rotational grazing by local herders and wild mushroom collection. The dominant understory shrubs include *Potentilla fruticosa*, *Potentilla glabra*, and *Caragana jubata*. Key herbaceous species consist of *Koeleria cristata*, *Pedicularis* spp., *Polygonum viviparum*, and *Carex atrata*. The soil types correlate with the vegetation zones, with altitudinal transitions from low to high elevations showing mountain forest gray-brown soils, mountain chestnut calcareous soils, meadow soils, subalpine shrub meadow soils, and alpine cold desert soils (Li et al., 2024). These soils are characterized by shallow profiles, coarse textures, predominantly silt loam, and parent materials including peatstone, conglomerate, and purplish-red sandy shale. Soil organic matter content is moderate, with a pH range of 7.0–8.0.

a: DEM map of the Qilian Mountains (red solid circles marking the Dayekou watershed); c: DEM map of the Dayekou watershed (red boxes delineating the boundary of the Pailugou watershed).

### 2.2 Data collection

#### 2.2.1 Remote sensing imagery data

**Figure 2:**
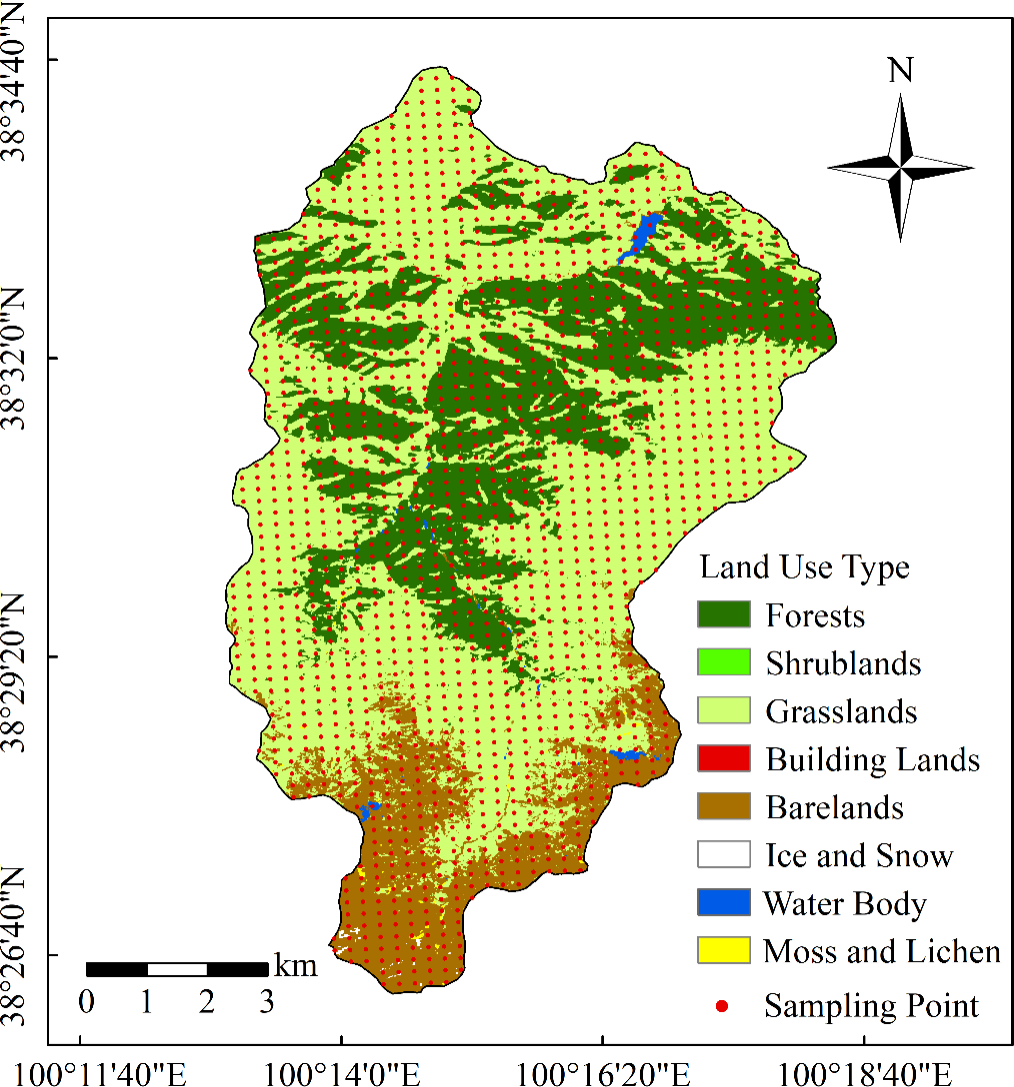
Distribution of topographic factor sampling plots in the Dayekou watershed.

Essential data were collected, including remotely sensed vegetation indices, land use information, and Digital Elevation Models (DEM) obtained from the Geospatial Data Cloud Platform (http://www.gscloud.cn), a public open-access repository hosted by the Computer Network Information Center of the Chinese Academy of Sciences. These data were derived from Sentinel-1C band synthetic aperture radar (SAR) echo distance imagery, which was fused with Sentinel-2 multispectral L2A-level surface reflectance imagery, resulting in a spatial resolution of 10 m. Initially, the DEM data were projected and registered using spatial masking based on the study area’s vector boundary file, followed by extent clipping to ensure full spatial coverage of the study area (Shao et al., 2019; Meroni et al., 2021; Pratama et al., 2024; Chen and Sun, 2024). ArcGIS 10.8 software was then used for vectorization analysis through the “Raster to Polygon” function, generating closed-topology polygonal vector surfaces. This transformation converted raster pixel features into vector surface features while preserving the original classification system attribute information, establishing a proper data framework for subsequent spatial statistical analysis (Bohn and Miller, 2024; Ma and Wang, 2024; Velamala and Pant, 2024). To precisely calculate the land area for each land use category, the “Calculate Geometry” function in GIS spatial analysis was employed, which automatically computed the projected area (in hectares) for each plot, creating an attribute-linked database. This allowed for accurate area statistics for each spatial unit, providing detailed unit area measurements for quantitative land use pattern analysis. For the extraction of topographic factors, the “Fishnet” function in ArcGIS 10.8 was used to extract DEM and other topographic factor raster data for the Dayekou watershed. A total of 1,785 sampling points were extracted at a 200 m sampling interval (**Figure 2**), including 503 points within Qinghai spruce forests and 1,282 points from non-forest land-cover types. During data processing, the “Slope” tool in ArcGIS Spatial Analyst was applied to the DEM data, generating terrain raster data, including slope, aspect, and latitude/longitude coordinates. Land use data were converted from raster to polygon vector format, and the vector extent of forested areas was extracted. Aspect data were then clipped based on this vector extent, and the data were reclassified into different aspect intervals. Finally, raster-to-polygon conversion was performed to obtain the forest area within each aspect interval (Congalton, 2022; Kang et al., 2024). In practice, the conifer-forest interpretation was cross-checked against historical forest compartment records for the watershed and field observations from representative stands at different elevations and slope aspects, and polygons requiring clarification were manually re-examined during interpretation.

To quantitatively assess the impact of slope aspect on the distribution of Qinghai spruce, aspect values were classified as follows: Plane: -1°; North (N): 0-22.5° and 337.5-360°; Northeast (NE): 22.5-67.5°; East (E): 67.5-112.5°; Southeast (SE): 112.5-157.5°; South (S): 157.5-202.5°; Southwest (SW): 202.5-247.5°; West (W): 247.5-292.5°; and Northwest (NW): 292.5-337.5°. For broader ecological interpretation, aspects were further grouped into shaded slopes (315-45°), semi-shaded slopes (45-135°), sunny slopes (135-225°), and semi-sunny slopes (225-315°).

#### 2.2.2 Meteorological data

The meteorological data used in this study were provided by the Qilian Mountains Forest Ecosystem Positioning Observation and Research Station. The data originate from a meteorological observation station located at an elevation of 2,570 m at the outlet of the Pailugou watershed (E 100°17′18″, N 38°34′1″). The dataset covers the period from January 1, 2003, to December 31, 2023. Situated at the watershed outlet, this station reflects regional climate trends and maintains continuous data over time. Calculations indicate that the study area at 2570 m recorded an average annual temperature of 1.77 °C and average annual precipitation of 384 mm for 2003-2023, with precipitation predominantly concentrated between June and September. Since the observation station is located at a lower elevation and the study plots are primarily distributed in the mid-mountain zone between 2700 and 3300 m, there is a notable elevation difference. The purpose of the elevation correction was to translate the station-based temperature and precipitation data at 2570 m to the main elevational range where Qinghai spruce occurs in the watershed. To ensure the representativeness of the meteorological data for the study area and to eliminate the potential impact of elevation differences on environmental factor analysis, elevation correction was applied to the data from the 2570 m site. This adjustment better aligns the data with the actual climatic conditions of the study plots. According to relevant studies, temperatures in the Qilian Mountains decrease with elevation at a rate ranging from approximately 0.58 °C/100 m to 0.66 °C/100 m, with specific values varying across different study areas and methodologies. Precipitation increases with elevation at a rate ranging from 18.6 mm/100 m to 27.7 mm/100 m, with an average increment rate of 4.99% (Zhao and Qian, 2025; Bai et al., 2023; Zhang et al., 2021). Based on these findings, which indicate an annual mean temperature of 1.77 °C and annual mean precipitation of 384 mm at the 2,570 m elevation, mathematical relationships between annual mean temperature (T), annual mean precipitation (P), and elevation changes were established:

(1) $$T=1.77-0.58\times \frac{H-2570}{100}$$

(2) $$P=384\times {\left(1+4.99\%\right)}^{\frac{H-2570}{100}}$$

In the formula, T represents the annual mean temperature (°C), P denotes the annual mean precipitation (mm), and H indicates the elevation (m). To better reflect the representativeness of the climatic data, we emphasize that the lapse-rate-corrected temperature and precipitation values provide first-order watershed-scale estimates rather than direct plot-level microclimatic observations. In mountainous terrain, microclimate may vary substantially among slope aspects and local topographic positions. Therefore, the climatic thresholds derived in this study should be interpreted as approximate distributional thresholds under the current interpolation scheme.

#### 2.2.3 Soil Survey

**Figure 3:**
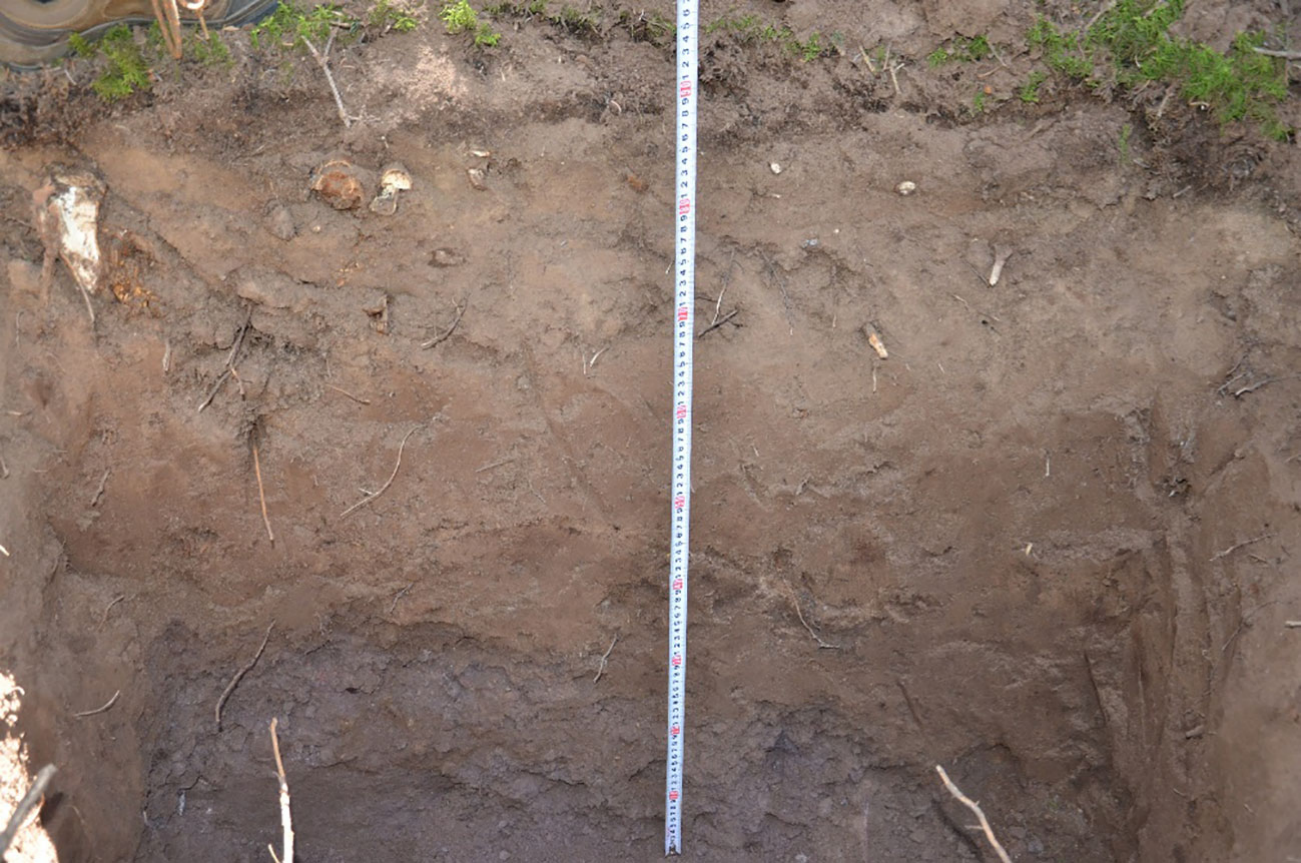
Soil profile survey photos of Qinghai spruce forests plots.

Currently, the accurate measurement of soil depth primarily relies on traditional physical methods. Digital Elevation Model (DEM) data, constrained by its surface attributes and resolution, cannot directly extract information from deeper soil layers (Zhang et al., 2017; Jafari et al., 2024). Consequently, this study employs the Soil Profile Method, which involves excavating and examining a vertical cross-section (profile) of the soil from the surface down to the parent rock. A total of sixteen 20 m × 20 m survey plots were established within the primary forest compartments of Qinghai spruce forests in the Pailugou watershed, at elevations ranging from 2700 to 3300 meters. For each plot, 3 to 5 sampling points were selected based on the diagonal rule—specifically at the start, end, and center along the plot’s diagonal. Soil profiles were excavated at these points until the parent rock layer was reached, forming a vertical cross-section. During excavation, the topsoil (A horizon), subsoil (B horizon), and substratum (C horizon) were clearly distinguished. Morphological characteristics, including color, texture, structure, and thickness, were recorded. Samples from the topsoil and substratum were collected for subsequent laboratory analysis of physical and chemical properties (Cools and Vos, 2013). Each profile was photographed to ensure accurate and clear representation (**Figure 3**). Following excavation, schematic sketches of the soil profiles were created, marking the boundaries, thicknesses, and notable features of each horizon. For the purposes of this study, only soil depth data were used in the analysis.

#### 2.2.4 Environmental factors

The environmental factors considered in this study included topographic (elevation, slope aspect, and slope gradient), climatic (precipitation and temperature), and soil factors (soil depth). The influence of these factors on the distribution of Qinghai spruce forests was analyzed through both univariate and multivariate statistical methods.

### 2.3 Statistical analysis

Statistical analysis was conducted to evaluate the relationship between Qinghai spruce distribution and various environmental factors. Initially, univariate statistical methods, including chi-square tests and analysis of variance (ANOVA), were used to assess the significance of topographic variables (elevation, aspect, and slope) and climatic factors (precipitation and temperature).

For multivariate analysis, decision tree and random forest models were used to quantify the relative importance of environmental factors influencing the distribution of Qinghai spruce forests. The decision tree model employed recursive partitioning to split the dataset according to the most informative variables (Ibrahim, 2023). For the decision tree analysis, the 1,785 sampling points extracted at 200 m intervals were randomly divided into training and testing sets, with 80% used for model training and 20% for testing. The maximum tree depth was set to 4, and the minimum number of samples per terminal node was set to 5 (Zhang et al., 2023). Model performance was evaluated using classification accuracy, precision, recall, and F1-score.

The random forest model was constructed using the same 1,785 sampling points and the same predictor variables (elevation, slope aspect, and slope gradient), with forest land coded as 1 and non-forest land coded as 0. Variable importance was derived from the fitted forest, and model performance was assessed using the out-of-bag (OOB) error and corresponding OOB accuracy. The remote-sensing-based area statistics reported were calculated from the complete classified land-cover polygons rather than from the 1,785 sampling points.

For reproducibility, the random forest was implemented in R with 500 trees, and variable importance was calculated as the mean decrease in Gini impurity from the fitted forest. Because the dataset contained 503 forest points and 1,282 non-forest points, no additional class weighting or re-sampling procedure was applied; instead, the moderate class imbalance was retained and considered when interpreting model performance.

Because the sampling points were extracted from continuous spatial layers within a single watershed, the 200 m spacing reduced local duplication but did not completely eliminate spatial autocorrelation. Therefore, the predictive performance of the decision tree and random forest models may be somewhat optimistic. Accordingly, the model results are interpreted primarily in terms of relative factor importance and broad ecological thresholds, rather than as strict measures of spatial prediction accuracy (Karabadji et al., 2023; Guhan and Revathy, 2024).

Data processing and statistical computations were carried out using ArcGIS 10.8 and R software. The charts were created using OriginPro 2024 and Excel. The significance level for all tests was set at p < 0.05.

## 3 Results

### 3.1 Distribution characteristics of Qinghai spruce

Based on remote sensing image analysis, the forest cover in the Dayekou watershed is approximately 27.93%, the remote sensing classification layer actually represents spruce-dominated coniferous woodland, rather than a strictly monospecific layer consisting solely of Qinghai spruce. Primarily consisting of Qinghai spruce and Qilian juniper. As both species are coniferous, remote sensing imagery cannot differentiate between them. Historical records and field surveys indicate that Qinghai spruce forests make up more than 95% of the total forest area in the watershed. Therefore, the potential areal overestimation caused by merging Qilian juniper into the conifer forest class is conservatively estimated to be less than 5% of the mapped woodland area (i.e., <1.01 km² of 20.28 km²). Qinghai spruce is distributed in patchy or linear formations on shaded and semi-shaded slopes between 2600 and 3400 meters in elevation, often interspersed with grasslands on sun-exposed slopes. The forest area in the Dayekou watershed spans approximately 20.28 km², while the grassland area covers about 42.93 km², accounting for the largest proportion at 59.03%. Statistics on the distribution of vegetation types in the Dayekou watershed are provided in **Table 1**.

**Table 1:** Area statistics of different land use types in the Dayekou watershed.

Land use type	Area(km^2^)	Percentage(%)
Grassland	42.93	59.03
Woodland	20.28	27.89
Shrubland	0.03	0.04
Bare ground/sparse vegetation	9.02	12.40
Buildings	0.01	0.01
Snow and ice	0.05	0.06
Water bodies	0.29	0.40
Mosses and lichens	0.12	0.16
Total	72.72	100.00

This interpretation was checked in practice against historical forest compartment information and field observations from representative conifer stands within the watershed, which consistently indicated that Qinghai spruce dominates the mapped conifer belt and that no extensive juniper-dominated patches were identified within the interpreted woodland polygons.

**Figure 4:**
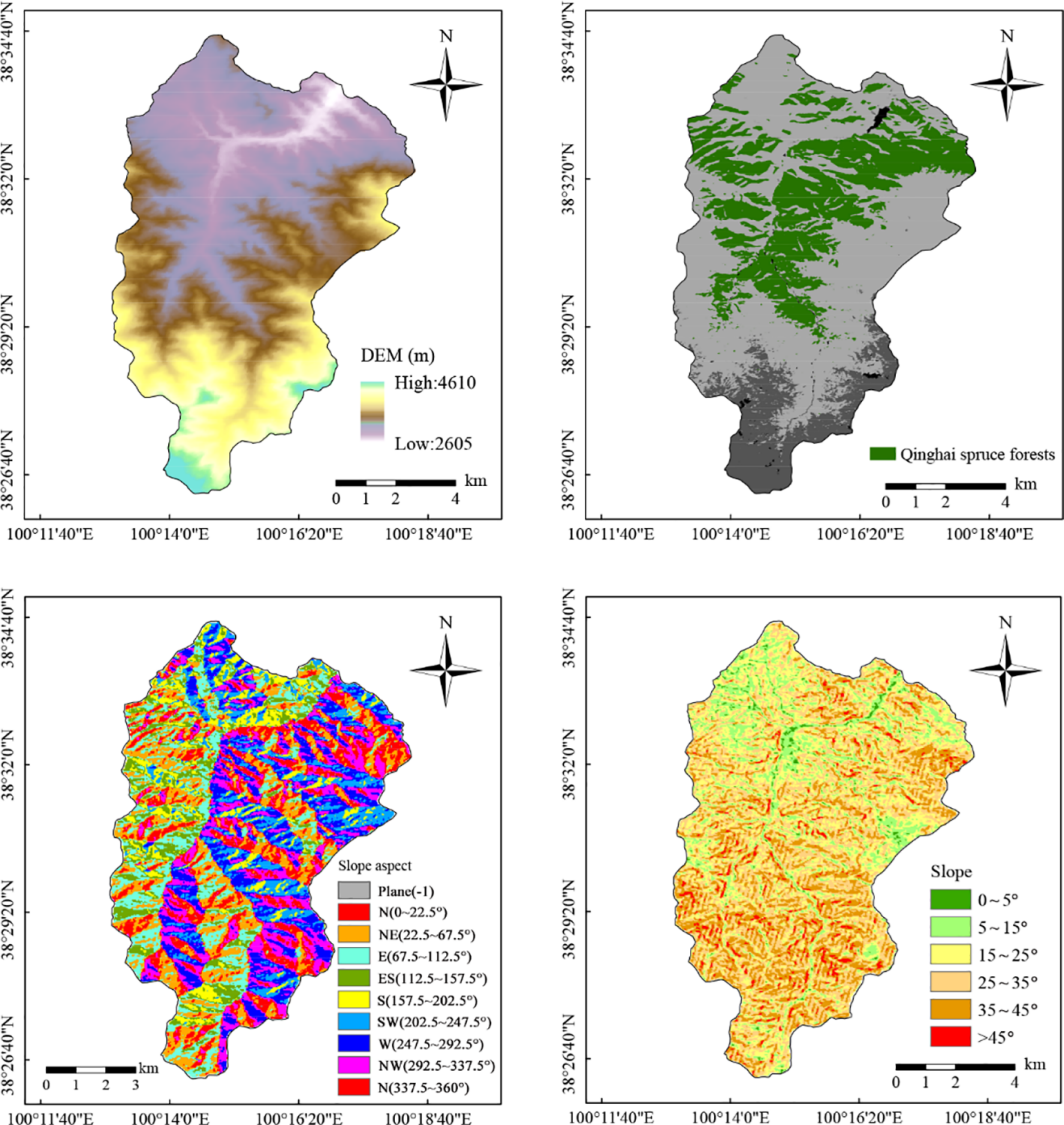
Spatial distribution patterns of DEM, Qinghai spruce forests patches, watershed aspect, and slope gradient in the Dayekou watershed.

From a spatial distribution perspective, Qinghai spruce forests are characterized by patchy and linear patterns (**Figure 4**), likely influenced by the region’s complex topography, soil conditions, and uneven water availability. These forest patches predominantly occur on shaded and semi-shaded slopes, accounting for 72.85% of the total forest patch area. An analysis of slope aspect distribution characteristics (**Table 2**) reveals that north (N), northeast (NE), and northwest (NW) orientations represent the primary distribution zones, collectively accounting for 69.73% of the total patch area.

**Table 2:** Area statistics of Qinghai spruce forests by aspect in the Dayekou watershed.

Serial no.	Slope aspect (aspect value)	Area of qinghai spruce forests(km^2^)	Percentage(%)
1	Plane: -1	0.07	0.34
2	N:0~22.5, 337.5~360	5.53	27.12
3	NE:22.5~67.5	5.40	26.48
4	E:67.5~112.5	1.95	9.56
5	ES:112.5~157.5	0.39	1.91
6	S:157.5~202.5	0.36	1.77
7	SW:202.5~247.5	0.92	4.51
8	W:247.5~292.5	2.47	12.11
9	NW:292.5~337.5	3.30	16.18

Based on slope distribution statistics for Qinghai spruce forests (**Table 3**), forest patches are predominantly concentrated within the 15~45° slope range, accounting for 85.72% of the total forest patch area. Along the elevation gradient, forest patches are primarily distributed between 2700 and 3300 meters, encompassing 97.5% of the total forest area.

**Table 3:** Area statistics of Qinghai spruce forests by slope gradient in the Dayekou watershed.

Serial no.	Slope(°)	Area of qinghai spruce forests(km^2^)	Percentage(%)
1	0~5	0.20	1.01
2	5~15	2.62	13.27
3	15~25	6.43	32.56
4	25~35	7.04	35.65
5	35~45	3.46	17.52

### 3.2 The influence of topographic factors on the distribution of qinghai spruce forests

**Figure 5:**
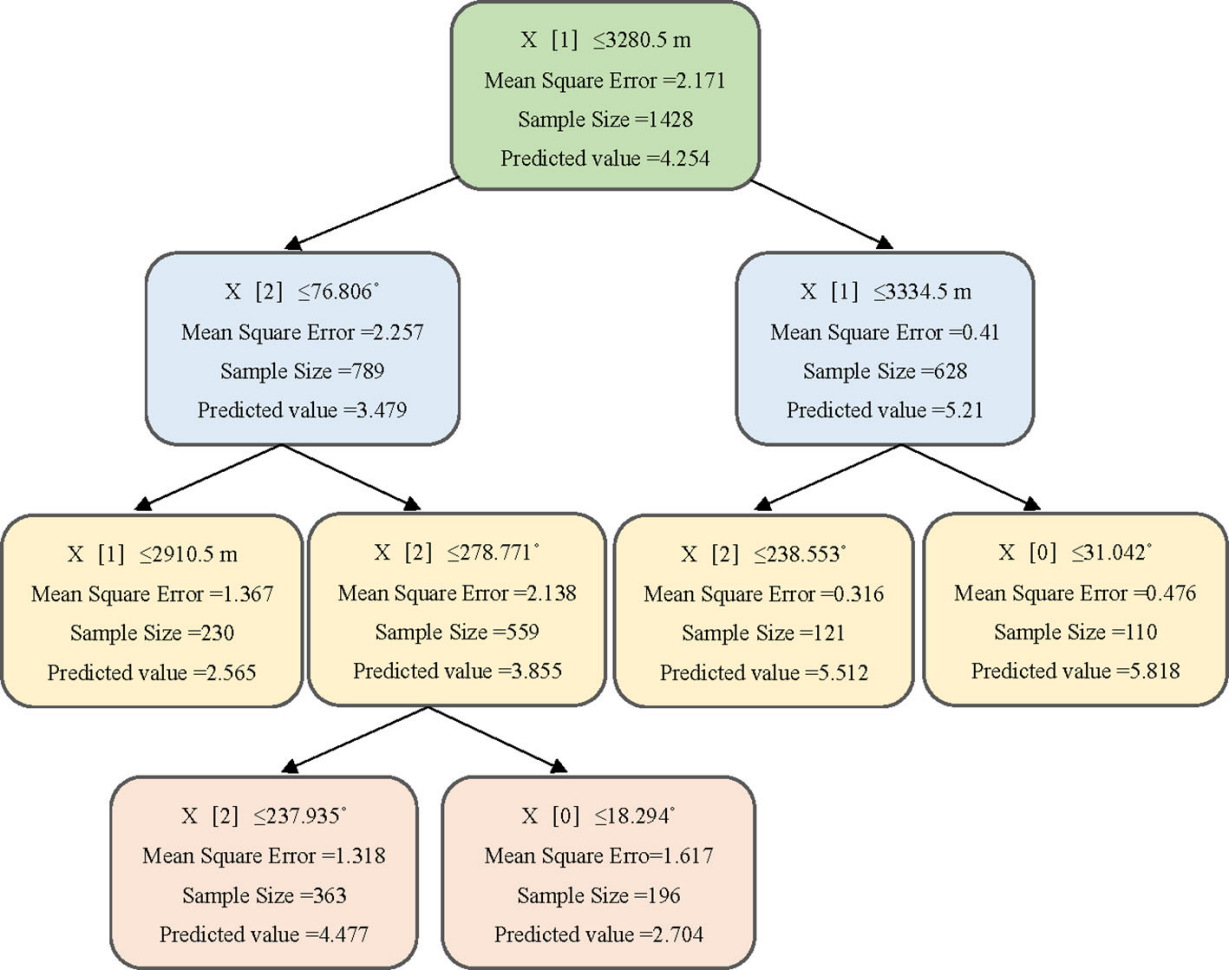
Decision tree model for predicting Qinghai spruce forests (Non-forest) distribution in the Dayekou watershed (Training Set Ratio = 0.8, X[1]: Elevation, X[2]: Aspect, X[0]: Slope).

Elevation, aspect, and slope data were extracted from a digital elevation model (DEM) for 1785 grid points within the study area. A decision tree model was constructed using elevation, aspect, and slope as independent variables and forested land (1) and non-forested land (0) as dependent variables to analyze the influence of topographic factors on the distribution of Qinghai spruce forests and non-forested areas in the Dayekou watershed. Model results (**Figure 5**) indicate that the root node classification occurs at an elevation of 3280.5 m, confirming elevation as the most significant topographic factor influencing Qinghai spruce forest distribution. At elevations ≤3280.5 m, samples were assigned to the left subtree and further classified by slope aspect (≤76.806°). At smaller slope aspects (≤76.806°), elevation remained the primary classification criterion, indicating that slope aspect significantly influences Qinghai spruce distribution in lower elevation zones. Ultimately, the model ceases splitting in areas with low slope aspect and elevation, delineating the optimal distribution range for Qinghai spruce. At elevations >3280.5 m, samples are assigned to the right subtree, where the first-level subnode uses elevation (≤3334.5 m) as the classification criterion. At higher elevations, slope aspect (≤238.6°) becomes a secondary classification factor, with further classification based on slope gradient (≤31.0°). This indicates that the combination of slope aspect and gradient plays a crucial role in the distribution of Qinghai spruce at higher elevations.

Overall, decision tree analysis reveals that elevation is the primary factor influencing the distribution of Qinghai spruce forests and non-forest areas. At elevations ≤3280.5 m, Qinghai spruce exhibits a higher probability of distribution, while at elevations >3280.5 m, the probability of non-forest areas increases. Slope aspect and gradient are secondary factors. Areas with a smaller slope aspect (≤238.6°) and gentler gradient (≤31.0°) are more conducive to Qinghai spruce distribution. Specifically, the distribution of Qinghai spruce forests is most favorable at an elevation of 2910.5 m.

Through a comparison of classification performance across decision tree models (**Table 4**), this model demonstrates strong overall classification effectiveness on the test set, achieving an accuracy rate of 87%. It performs particularly well in classifying non-forest land (0), with a precision of 88%, recall of 92%, and an F1-score of 0.90. This indicates that the model has high recognition capability and accuracy for non-forest land samples. Its performance in classifying forest land (1) is slightly weaker, with a precision of 77%, recall of 68%, and an F1-score of 0.72. This suggests that the model has some limitations in identifying forest land samples, potentially missing some of them. Additionally, due to the imbalanced distribution of test set samples, with non-forest samples vastly outnumbering forest samples, the model may exhibit a stronger preference for non-forest areas. Overall, the model demonstrates high accuracy in classifying both forest and non-forest areas.

**Table 4:** Performance comparison of decision tree models on the test dataset.

Category	Precision	Recall	F1-Score	Support
Non-forest land (0)	0.88	0.92	0.90	255
Forest land (1)	0.77	0.68	0.72	102

**Figure 6:**
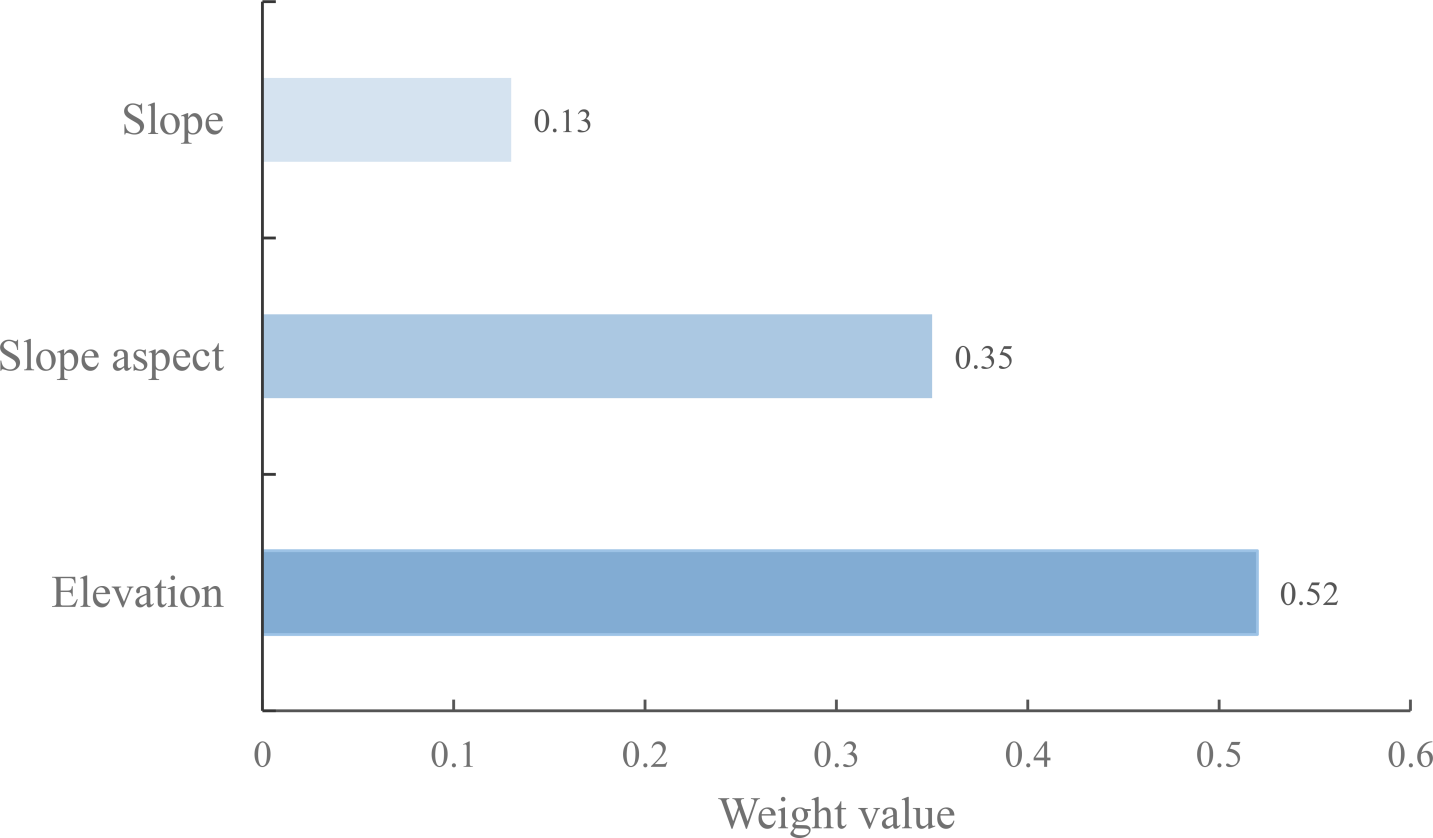
Feature importance plot of topographic factors influencing the distribution of Qinghai spruce.

Using slope gradient, elevation, and slope aspect as predictor variables and land-cover type (forest/non-forest) as the response variable, the random forest model showed good overall classification performance. The OOB accuracy was 87.17%, corresponding to an OOB error of 12.83%. A 10-fold cross-validation further produced a mean accuracy of 87.17% (SD = 2.83%). The results (**Figure 6**) indicate that elevation is the most significant factor influencing the distribution of Qinghai spruce forests, with a weight of 0.52. This highlights the substantial role of elevation in determining the presence or absence of Qinghai spruce forests. Slope aspect was ranked as the second most important factor, with a weight of 0.35, suggesting that slope orientation also plays a significant role in Qinghai spruce forest distribution. Slope gradient had the lowest influence weight at 0.13, indicating its relatively minor impact on the distribution of Qinghai spruce forests.

**Figure 7:**
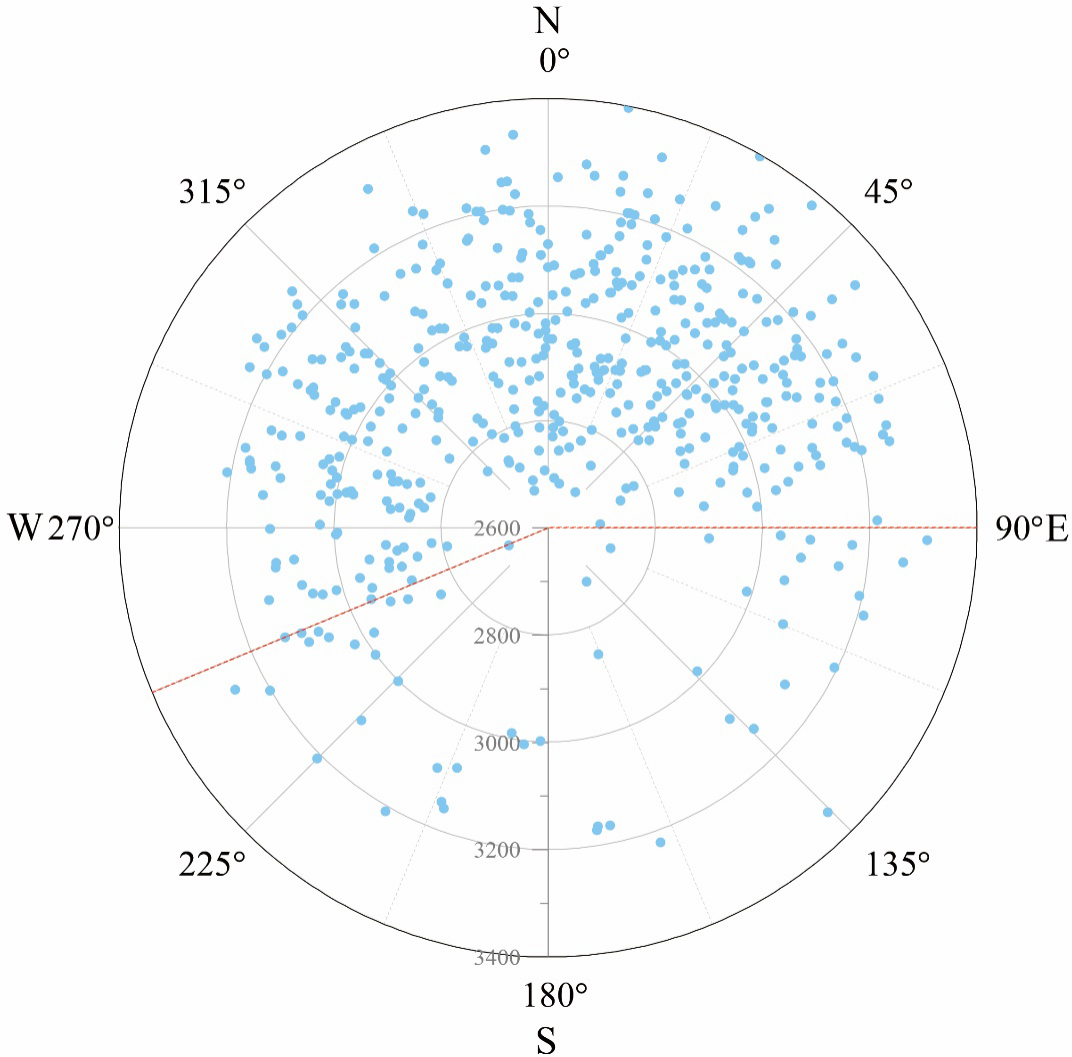
Relationship between Qinghai spruce forests distribution and elevation/slope aspect in the Dayekou watershed (The red line in the upper portion of the plot indicates the area where Qinghai spruce forest distribution is concentrated).

According to the results of the random forest analysis, elevation and slope aspect are the two most significant factors influencing the spatial distribution of Qinghai spruce. By extracting elevation and slope aspect information from forest grid data points in the remote sensing data of the Dayekou watershed, these factors were used as the radial and angular axes, respectively, to plot a polar coordinate diagram (**Figure 7**). In this plot, the position of each data point is determined by its corresponding elevation and slope aspect values. The elevation value dictates the radial distance from the origin, while the slope aspect value determines the angular position relative to the polar axis. This diagram illustrates the relationship between Qinghai spruce distribution and elevation/slope aspect in the Dayekou watershed. **Figure 6** shows that Qinghai spruce occurs at elevations between 2626 and 3398 m, with the majority distributed between 2900 and 3300 m. Its distribution is most extensive near 3000 m, indicating that the population primarily inhabits this elevation range—a finding that aligns well with field surveys. Analysis of slope aspect data within the Qinghai spruce distribution zone reveals that the species predominantly inhabits shaded or semi-shaded slopes with aspects greater than 240° or less than 90°. Ninety-five percent of the distribution occurs within the 0–90° and 240–360° aspect ranges. As elevation increases and precipitation rises, the range of slope orientations where Qinghai spruce occurs also expands. At elevations between 2900 and 3300 m, the distribution of Qinghai spruce across slope orientations is most extensive. Beyond this elevation, as elevation increases, the range of slope orientations where Qinghai spruce occurs narrows.

**Figure 8:**
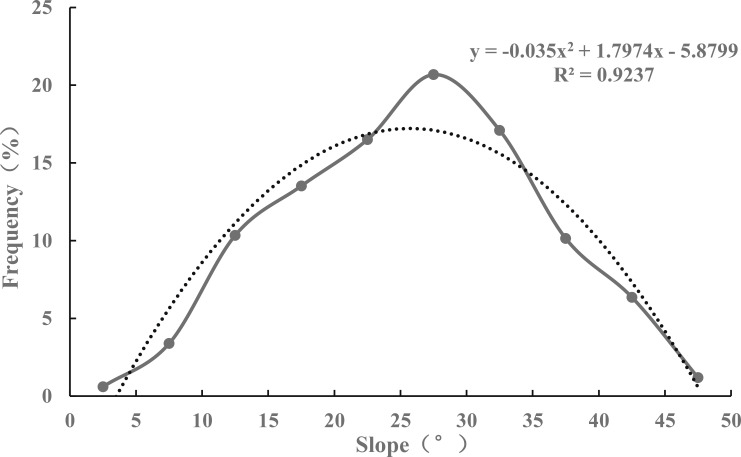
Relationship between distribution frequency of Qinghai spruce and slope gradient.


Slope can influence forest distribution by affecting factors such as solar radiation, soil texture, soil erosion, soil moisture, and nutrient conditions. However, based on decision tree analysis, the third decision node for slope classification occurs at <24.9°, indicating that slope has a relatively minor influence on Qinghai spruce distribution. Therefore, slope is not a limiting factor for Qinghai spruce forest distribution. In this study, Qinghai spruce forests are distributed across slopes ranging from 3.8° to 49.7°, with an average slope of 26.06°. Analysis of the relationship between Qinghai spruce distribution zones and slope gradients reveals the distribution frequency of Qinghai spruce across different slope levels (**Figure 8**) Frequent occurrences are concentrated between 12° and 38°, with the highest distribution frequency occurring between 25° and 30°. Furthermore, 85% of Qinghai spruce forests grow on medium or steep slopes exceeding 15°. Correlation function fitting determined that the distribution frequency of Qinghai spruce forests in the study area best fits a binomial function, yielding the highest R² value. The relationship is expressed as:

$$\text{y }=\;-0.35{\text{x}}^{2}\;+\;1.7974\text{x }-\;5.8799$$

In the equation, y represents the distribution frequency of Qinghai spruce, x denotes slope, and the correlation coefficient (R^2^) is 0.92.

### 3.3 Effects of temperature and moisture on the distribution of Qinghai Spruce

The distribution of mountain forests in arid regions is influenced by numerous climatic factors, including temperature, precipitation, and solar radiation. Among these, annual mean temperature and annual mean precipitation are the most significant and readily available, making them the two most frequently utilized variables in previous studies (Hending et al., 2022). Typically, as elevation increases, precipitation rises while temperatures decrease, reducing evapotranspiration and increasing water availability for plants. This significantly affects the distribution of high-elevation mountain forests.

**Figure 9:**
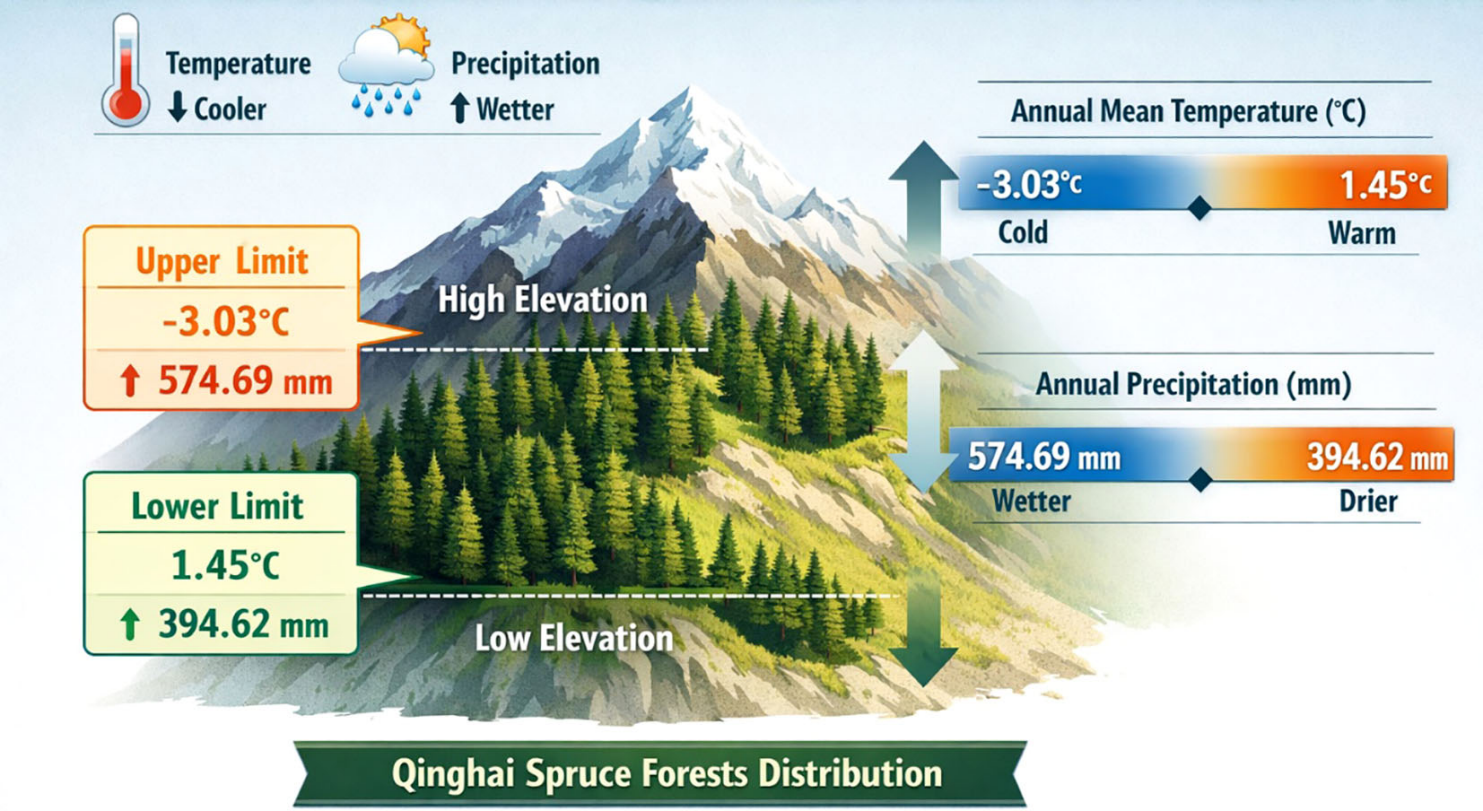
Climatic Limits of Qinghai 0.

Using the elevation-corrected temperature and precipitation relationships (Equations 1, 2), we estimated the broad climatic envelope associated with the observed distribution of Qinghai spruce in the study area. Under the present interpolation framework, the upper and lower distribution limits correspond to annual mean temperatures of approximately -3.03 °C and 1.45 °C, respectively, while the associated annual precipitation thresholds are approximately 574.69 mm and 394.62 mm (**Figure 9**). These values should be interpreted as approximate watershed-scale occurrence thresholds rather than precise microclimatic or physiological limits, because local hydrothermal conditions may vary among slope aspects and topographic positions.

### 3.4 Influence of soil depth on the distribution of Qinghai spruce

**Figure 10:**
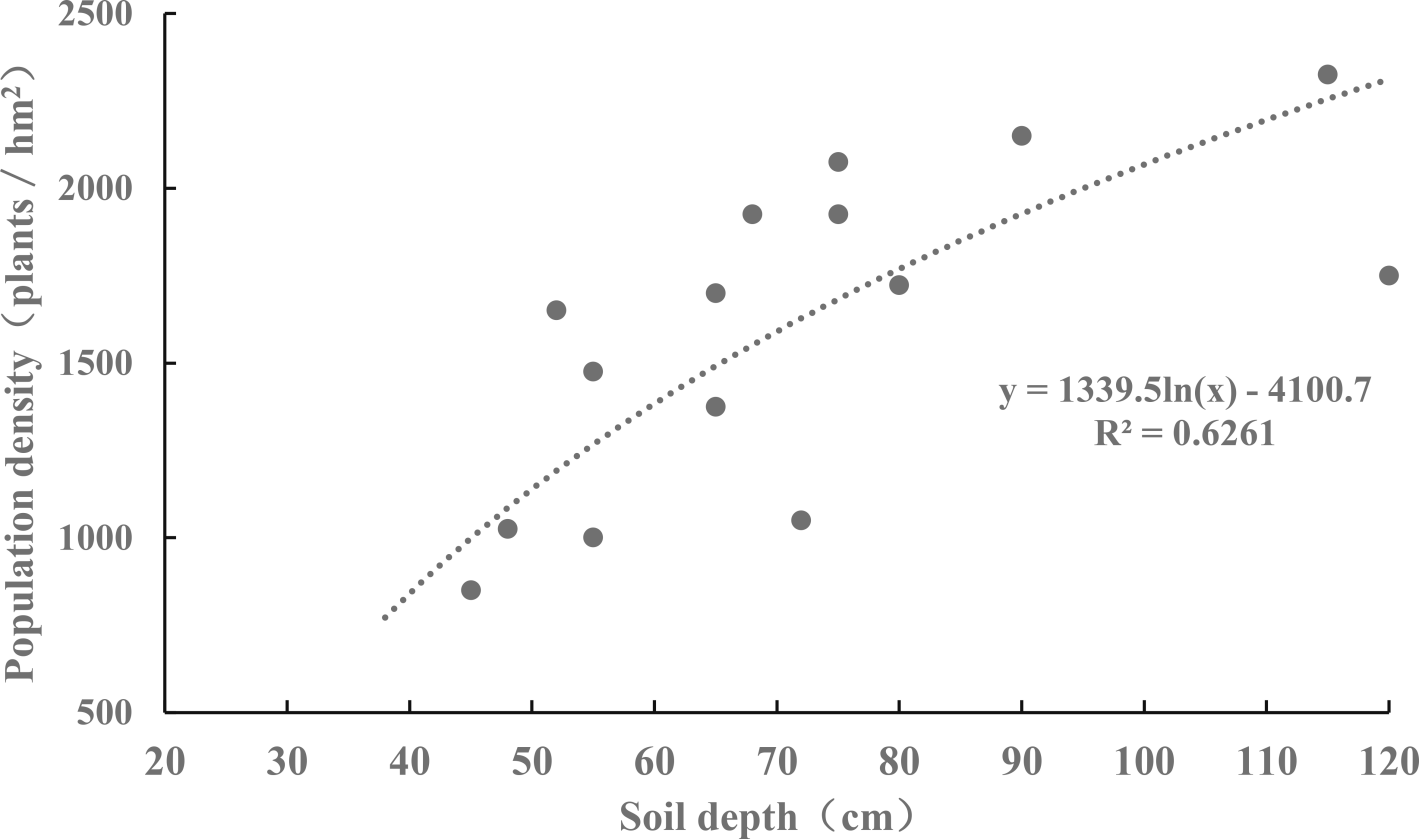
Variation in population density of Qinghai spruce forests with soil depth.

Deep soil layers buffer external environmental changes, enhancing Qinghai spruce’s resistance to adverse conditions. In contrast, areas with shallow soil layers exhibit weaker buffering capacity, potentially making the spruce more susceptible to environmental stressors. Although this study could not comprehensively address all forest soil conditions due to limitations, investigations of soil profiles at different altitudinal gradients across 15 Qinghai spruce survey plots in the Pailugou watershed revealed significant variation in soil depth. Soil depths ranged from 30 cm to 120 cm, with greater soil depth at lower elevations, gradually decreasing with increasing elevation. Near the upper limit of Qinghai spruce distribution (3300 m elevation), soil depth is only 30–40 cm. For the soil-depth analysis shown in **Figure 10**, population density was calculated as the number of Qinghai spruce individuals recorded within each 20 m × 20 m plot and then converted to plants per hectare. Based on plot soil profile surveys, Qinghai spruce population density exhibits logarithmic growth with increasing soil depth. Specifically, population density is low in shallow soils (30–50 cm), where root restriction is the primary limiting factor. Population density increases significantly in deeper soil layers (above 80 cm), suggesting that deeper soils may provide a more stable water and nutrient supply, fostering population development and growth. The R2 value of the logarithmic function fit is 0.6261, indicating a moderate degree of fit between soil depth and Qinghai spruce population density.

## 4 Discussion

### 4.1 Main findings and ecological mechanisms

This study shows that the distribution of Qinghai spruce in the Dayekou watershed is controlled primarily by elevation and secondarily by slope aspect, whereas slope gradient plays a weaker role. Rather than acting as purely geometric terrain variables, elevation and aspect function mainly as integrated controls on local hydrothermal conditions. The observed concentration of Qinghai spruce between 2900 and 3300 m and on shaded to semi-shaded slopes indicates that the species occupies habitats where temperature and moisture are balanced most favorably for survival, regeneration, and stand persistence (Niu et al., 2024; Li et al., 2024).

The dominant effect of elevation can be interpreted as the result of multiple coupled ecological processes. Along the elevational gradient, temperature generally decreases, precipitation tends to increase, and evaporative demand is reduced. In the lower part of the watershed, warmer conditions and stronger evapotranspiration likely intensify soil moisture limitation, thereby restricting Qinghai spruce establishment and persistence. By contrast, at higher elevations, low temperature, a shorter growing season, stronger freeze–thaw stress, and shallower soils may jointly constrain tree growth and contribute to the upper distribution limit. Thus, the elevational range of Qinghai spruce in this watershed likely reflects a trade-off between drought stress at lower elevations and cold limitation at higher elevations, with the middle belt representing the most suitable hydrothermal niche (Tian et al., 2024; Hou et al., 2025).

Slope aspect further modifies this elevational effect by regulating incoming solar radiation, snow retention, soil evaporation, and near-surface moisture conditions. In semi-arid mountain systems such as the Qilian Mountains, north-, northeast-, and northwest-facing slopes generally experience lower radiation loads and reduced evaporative loss, allowing more favorable soil water conditions for conifer establishment and persistence. This helps explain why Qinghai spruce is concentrated on shaded and semi-shaded slopes (Zhan et al., 2025). Aspect therefore does not act independently of climate, but instead mediates how the regional climate signal is expressed at the microsite scale. In this sense, aspect can be understood as a key topographic filter that locally amplifies or buffers hydrothermal stress (Malone et al., 2025; McNichol et al., 2024).

By comparison, slope gradient appears to have a weaker direct effect on spruce occurrence. Its ecological role is more likely indirect, through influences on runoff, infiltration, erosion, soil thickness, and substrate stability. In the present study, slope gradient contributed less than elevation and aspect, suggesting that it is not the principal limiting factor at the watershed scale. However, it may still influence local stand structure and regeneration through its interaction with soil depth, water redistribution, and microtopographic heterogeneity (Malone et al., 2025).

### 4.2 Comparison with previous studies

The present results are broadly consistent with previous studies showing that topography and climate jointly govern the distribution of Qinghai spruce in the Qilian Mountains. Earlier work has emphasized the importance of elevation, moisture availability, and slope aspect in shaping both adult forest distribution and seedling recruitment along mountain gradients (Huang et al., 2024; Liu et al., 2025). Our results support this general pattern and further indicate that, at the watershed scale, elevation and aspect act as the most effective terrain-based proxies for the hydrothermal environment experienced by Qinghai spruce.

At the same time, this study contributes a somewhat different perspective from plot-based or physiological studies. Many previous studies on Qinghai spruce and similar montane conifers have focused on direct climate–growth relationships, such as radial growth responses to temperature, precipitation, drought, and vapor pressure deficit. Those studies help explain how climatic variability affects tree performance over time (Zhan et al., 2025; Liu et al., 2024a; Hou et al., 2026). In contrast, our analysis addresses spatial distribution patterns within a single watershed and therefore identifies the terrain factors that integrate long-term climatic constraints across the landscape. In this context, the strong role of elevation and aspect does not contradict climate-based studies; rather, it indicates that topography is the main spatial framework through which climatic constraints are organized at the local scale (Das et al., 2015; Shukla et al., 2023).

Compared with earlier threshold-based studies in the Qilian Mountains, our results are also broadly similar in identifying the mid-elevation belt and shaded aspects as the most favorable habitats for Qinghai spruce (Yang et al., 2017). However, the present study extends previous work by combining full-coverage remote-sensing-based forest mapping with machine-learning analysis of watershed-scale samples. This combination allows the relative contribution of terrain variables to be evaluated more explicitly and provides a spatially continuous interpretation of habitat suitability. At the same time, our results suggest that the apparent strength of topographic drivers should be interpreted cautiously, because these drivers partly represent underlying climatic and hydrological processes rather than purely independent controls (Yang et al., 2026; Liu et al., 2024b).

### 4.3 Implications of climatic thresholds under climate change

The temperature and precipitation ranges identified in this study are useful for describing the approximate climatic window within which Qinghai spruce currently occurs in the Dayekou watershed. Under ongoing climate warming, these thresholds have important ecological implications. In principle, warming may relax low-temperature constraints near the upper elevational limit, potentially allowing spruce occurrence to extend upward where soil and substrate conditions remain suitable. However, warming may simultaneously intensify drought stress at the lower elevational limit and on sunnier slopes by increasing evapotranspiration and atmospheric water demand. As a result, future distribution shifts may be asymmetric: the lower and drier margins of the species may become more vulnerable than the upper and shaded margins (Foti and Ramírez, 2013; Andrew et al., 2016).

This interpretation is especially relevant in arid and semi-arid mountain regions, where forest persistence is often controlled less by absolute temperature alone than by the balance between temperature and plant-available moisture. In such systems, north-facing and mid-elevation habitats may serve as relative refugia because they buffer heat and moisture stress more effectively than south-facing or lower-elevation sites (Pepin et al., 2025; Hou et al., 2026). Therefore, the climatic thresholds identified here should not be viewed as fixed physiological boundaries, but rather as watershed-scale occurrence ranges that reflect the current interaction among topography, temperature, and moisture.

These findings also have practical implications for afforestation and ecological restoration. Under future warming scenarios, priority areas for Qinghai spruce restoration should likely remain concentrated in shaded to semi-shaded slopes within the mid-elevation belt, where hydrothermal conditions are relatively stable. Conversely, lower-elevation and sunnier sites may face increasing establishment risk and should be evaluated more cautiously, especially where soil depth is limited or water deficits are already pronounced.

### 4.4 Limitations and uncertainties

Several limitations should be acknowledged when interpreting the results of this study. First, the climatic analysis is based on data from a single meteorological station located at 2570m, and temperature and precipitation at higher elevations were estimated using literature-based lapse rates. Although this approach is common in mountain studies and provides a practical first-order approximation, it cannot fully capture the strong microclimatic heterogeneity of complex terrain (Marta et al., 2023; Pepin et al., 2025). In particular, slope aspect, cold-air drainage, local shading, and topographic exposure may all produce climate deviations that are not represented by a single station or by static elevation corrections. Therefore, the climatic thresholds reported here should be interpreted as approximate watershed-scale occurrence thresholds rather than precise microclimatic limits.

Second, the forest area statistics derived from remote sensing represent spruce-dominated coniferous woodland rather than a strictly monospecific Qinghai spruce layer. Because Qinghai spruce and Qilian juniper cannot be completely separated in the current classification, some uncertainty remains in the estimated forest area and in the ecological interpretation of the distribution pattern, particularly in habitats where the two species may co-occur. Although field evidence suggests that Qinghai spruce strongly dominates the mapped conifer belt, this limitation should still be recognized.

Third, the decision tree and random forest analyses were based on 1,785 sampling points extracted from continuous spatial layers within a single watershed. Although the 200 m point spacing reduces local duplication, it does not fully eliminate spatial autocorrelation. As a result, model performance may be somewhat optimistic, and the classification results should be interpreted mainly in terms of relative factor importance and broad ecological thresholds rather than strict spatial prediction accuracy.

Finally, this study focuses mainly on topographic, climatic, and soil-depth variables, while other potentially important drivers—such as soil moisture dynamics, snowpack duration, disturbance history, grazing pressure, and biotic interactions—were not explicitly incorporated into the models. Future studies should integrate plot-level microclimate observations, species-specific classification methods, and spatially explicit validation strategies to refine the ecological thresholds and improve mechanistic understanding of Qinghai spruce distribution in mountain environments (Malone et al., 2025; McNichol et al., 2024; Pepin et al., 2025).

## 5 Conclusions

This study used remote sensing data, topographic analysis, climatic extrapolation, and field soil surveys to examine the environmental controls on the distribution of Qinghai spruce forests in the Dayekou watershed. Because remote sensing imagery could not fully separate Qinghai spruce from Qilian juniper, the mapped woodland layer should be interpreted as spruce-dominated coniferous forest, which introduces limited uncertainty into the area estimates. Within this watershed, Qinghai spruce occurred mainly on shaded to semi-shaded slopes (aspects >240° or <90°) and on medium to relatively steep slopes (>15°). Its broadest distribution was observed between 2900 and 3300 m, and the most favorable occurrence zone identified by the decision tree was near 2910.5 m, with smaller aspect values (<=238.6°) and gentler slopes (<=31.0°) being associated with higher occurrence probability. Elevation was the most important topographic factor, followed by slope aspect, whereas slope gradient played a weaker role. Under the present elevation-correction framework, the observed distribution corresponded to an approximate annual mean temperature range of -3.03 °C to 1.45 °C and an annual precipitation range of 394.62-574.69 mm. These values, as well as the elevation, aspect, and slope ranges identified in this study, should be regarded as study-area-supported occurrence thresholds rather than universally fixed criteria for all mountain ecosystems. Nevertheless, they provide practical guidance for afforestation and ecological restoration in the Qilian Mountains, where priority sites are likely to be north-, northeast-, and northwest-facing slopes with slope angles of about 25-30° and elevations of about 2700–3300 m.

## Data Availability

The original contributions presented in the study are included in the article/supplementary material. Further inquiries can be directed to the corresponding author.
